# Efficiency of ventilators for intermediate care to deliver adequate FI02. a bench study

**DOI:** 10.1186/2197-425X-3-S1-A269

**Published:** 2015-10-01

**Authors:** L Baboi, S Guegan, C Guérin

**Affiliations:** Hôpital de la Croix Rousse, Réanimation Médicale, Lyon, France; Ecole des Haute Etudes Ingenieur, Lille, France; INSERM UMR 955, Créteil, France

## Introduction

There is an ongoing development of turbine powered ventilators able to support patients with mild acute respiratory failure in the intermediate care environment at the hospital. The capability for these home care ventilators to deliver adequate FI02 is an important end-point for patient's safety.

## Objectives

To compare on the bench the performance of ventilators for intermediate care to deliver appropriate FI02 when non vented (double limb) circuit was used.

## Methods

Astral 150, Elisée 150, Trilogy 200 and Monnal T50 equipped with non-vented double limb circuit or equivalent were set in volume controlled mode (tidal volume 500 ml, breathing frequency 15 cycles/min, inspiratory time 0.8 sec, constant flow inflation shape, positive end expiratory pressure 5 cmH2O) and connected to ASL 5000 lung model set in a condition mimicking COPD patient (compliance 75 ml/cmH2O, inspiratory and expiratory airways resistance 15 and 25 cmH2O/L/s, respectively). Oxygen was supplemented by connecting the low pressure oxygen port at the rear of ventilator to the wall oxygen supply via a flow-meter. Three oxygen flow rates were delivered: 0, 3 and 15 L/min. FI02 was measured with Citrex H4™ (imtmedical, Switzerland) and continuously monitored at the screen of the device. Once FI02 reached a plateau oxygen administration was maintained for an additional one minute and FI02 was measured over the last 10 breaths of the recording at the steady state defined as changes in FI02 less than 10% from the previous sampling window. The sampling rate was 200 Hz. Citrex H4 ™ device was calibrated before each experiment. The values of FI02 were expressed as mean ± SD. Comparisons were made by using two-factor ANOVA and multiple comparisons between ventilators by using Tukey test.

## Results

The values of FI02 are shown in the table. There is a significant effect of both oxygen flow rate supplementation and ventilator (P < 0.001 for each) and a significant interaction between them (P < 0.001). For any given level of oxygen flow rate supplementation all differences between multiple comparisons across ventilators were statistically significant.

**Table 1:** Table 1

	Oxygen supplementation		
	0 L/min	3 L/min	15 L/min
Astral 150	22 ± 0.0	42 ± 0.0	99 ± 0.0
Elisée 150	21 ± 0.0	35 ± 0.0	94 ± 0.0
Monnal T 50	21 ± 0.0	40 ± 0.0	86 ± 0.0
Trilogy 200	22 ± 0.0	30 ± 0.0	62 ± 0.0

## Conclusions

Some ventilators were associated with significantly higher levels of FI02 than the others at any rate of oxygen supplementation. At 15 L/min oxygen supplementation, FI02 was close to 100% with the Astral ventilator. These results might open some new opportunities, such as allowing walking even for intubated hypoxemic patients in sub-acute facilities, especially when devices offer good mobility and Sp02 monitoring.

